# Association between coffee and green tea intake and pneumonia among the Japanese elderly: a case-control study

**DOI:** 10.1038/s41598-021-84348-w

**Published:** 2021-03-10

**Authors:** Kyoko Kondo, Kanzo Suzuki, Masakazu Washio, Satoko Ohfuji, Satoru Adachi, Sakae Kan, Seiichiro Imai, Kunihiko Yoshimura, Naoyuki Miyashita, Nobumitsu Fujisawa, Akiko Maeda, Wakaba Fukushima, Yoshio Hirota, The Pneumonia in Elderly People Study Group, Kanzo Suzuki, Masakazu Washio, Kyoko Kondo, Satoko Ohfuji, Akiko Maeda, Wakaba Fukushima, Yoshio Hirota, Satoru Adachi, Sakae Kan, Seiichiro Imai, Kunihiko Yoshimura, Naoyuki Miyashita, Nobumitsu Fujisawa, Noriko Kojimahara, Chiharu Ota, Ikuji Usami, Munehiro Kato, Toshinobu Yamamoto, Kazuhide Yamamoto, Yoichi Nakanishi, Takanari Kitazono, Takafumi Matsumoto, Hideki Tashiro, Masahiko Taketomi, Tomoaki Iwanaga, Hiroko Nogami, Koichi Takano, Ken Tonegawa, Yoshimitsu Hayashi, Ikuo Ikeda, Shigeki Sugiyama, Masahiro Aoshima, Kei Nakashima, Yoshitaka Nakamori, Yasushi Seida, Yoshiko Kichikawa, Atsushi Nakamura, Yasuhito Iwashima, Yasuhiro Kojima, Yasuo Yamada, Hidekazu Kawamura, Toshiaki Niwa, Atsuro Kawai, Yuuji Ito, Emi Aoyama, Noriko Kusada, Chizuko Sumida

**Affiliations:** 1 Management bureau, Osaka City University Hospital, 1-5-7 Asahi-machi, Abeno-ku, Osaka, 545-8586 Japan; 2 Department of Community-Based Medical Education, Nagoya City University Graduate School of Medical Sciences, Nagoya, Japan; 3 School of Nursing, Nagoya City University, Nagoya, Japan; 4 Department of Community Health and Clinical Epidemiology, St. Mary’s College, Kurume, Japan; 5 Department of Public Health, Osaka City University Graduate School of Medicine, Osaka, Japan; 6 Research Center for Infectious Disease Sciences, Osaka City University Graduate School of Medicine, Osaka, Japan; 7 Department of Pulmonology, Kasadera Hospital, Nagoya, Japan; 8 Kaisei Hospital, Nagoya, Japan; 9 Department of Respiratory Medicine, Kyoto University Hospital, Kyoto, Japan; 10 Preemptive Medicine and Lifestyle Related Disease Research Center, Kyoto University Hospital, Kyoto, Japan; 11 Department of Pulmonology, Mitsui Memorial Hospital, Tokyo, Japan; 12 Department of Internal Medicine, Kawasaki Medical School Hospital, Okayama, Japan; 13 St. Mary’s Hospital, Kurume, Japan; 14 Clinical Epidemiology Research Center, Medical Co. LTA (SOUSEIKAI), Fukuoka, Japan; 15 College of Healthcare Management, Miyama, Japan; 16 Tokyo Women’s Medical University, Tokyo, Japan; 17 Asahi Rosai Hospital, Owariasahi, Japan; 18 Kazu Clinic, Toyohashi, Japan; 19 Graduate School of Medical Sciences, Kyushu University, Fukuoka, Japan; 20 Doukai Clinic, Ookawa, Japan; 21 Fukuoka National Hospital, Fukuoka, Japan; 22 Nishifukuoka Hospital, Fukuoka, Japan; 23 Nagoya City Koseiin Geriatric Hospital, Nagoya, Japan; 24 Kasugai Municipal Hospital, Kasugai, Japan; 25 Ikeda Clinic, Nagareyama, Japan; 26 Sugiyama Clinic, Susono, Japan; 27 Kameda Medical Center, Kamogawa, Japan; 28 Mishuku Hospital, Tokyo, Japan; 29 Nagoya City University Graduate School of Medical Sciences, Nagoya, Japan; 30 Iwashima Clinic, Mizunami, Japan; 31 Kojima Clinic, Nagoya, Japan; 32 Yama Clinic, Aichi, Japan; 33 Kawamura Clinic, Seki, Japan; 34 Hamada Asai Clinic, Tajimi, Japan; 35 Kawai Clinic, Osaka, Japan; 36 Daiyukai Hospital, Ichinomiya, Japan; 37 Inazawa Municipal Hospital, Inazawa, Japan

**Keywords:** Medical research, Risk factors

## Abstract

A large prospective cohort study in the United States examined the association between coffee intake and overall and cause-specific mortality and showed a inverse association between pneumonia and influenza deaths and coffee intake. In Japan, the mortality rate of pneumonia in elderly people is high, and its prevention is an important issue. The present study investigated the association between coffee and green tea intake and pneumonia among the elderly. The design was a hospital-based case control study. The cases were patients over 65 years old newly diagnosed as pneumonia. As a control, patients with the same sex and age (range of 5 years) who visited the same medical institution around the same time (within 2 months after examination of the case) for a disease other than pneumonia were selected. There were two controls per case. Odds ratio (OR) and 95% confidence interval (CI) for pneumonia of coffee and green tea intake during the past month were calculated using a conditional logistic regression model. A total of 199 cases and 374 controls were enrolled. When compared to those who do not drink coffee, the OR for pneumonia of those who drink less than one cup of coffee per day was 0.69 (95% CI 0.39–1.21), OR of those who drink one cup was 0.67 (0.38–1.18), and OR of those who drink two or more cups was 0.50 (0.28–0.88) (Trend p = 0.024). No association was found between pneumonia and green tea consumption. This study suggested a preventive association between coffee intake over 2 cups per day and pneumonia in the elderly.

## Introduction

Coffee has been consumed all over the world, and many studies are considering its impact on health^1,2^. A large prospective cohort study in the United States has shown an inverse association between coffee intake and all-cause mortality, and in cause-specific deaths, coffee intake has inversely associated with heart disease, chronic respiratory disease, diabetes, pneumonia and influenza, and suicide^3^. A cohort study in Japan also showed an inverse association with coffee intake and overall mortality, cause-specific death by heart disease, cerebrovascular disease and respiratory disease^4^. Green tea intake was inversely associated with overall mortality and cause-specific death by heart disease, and it was inversely associated with cause-specific death by cerebrovascular disease and respiratory disease in men^5^.

In Japan, elderly people have high age-specific mortality rates of pneumonia, especially high age at 80 years and over^6^. Because Japan is aging at a pace unparalleled in other countries, the prevention of elderly person pneumonia is an important problem. Epidemiological studies on pneumonia in the elderly have been studied on the effectiveness of vaccination (streptococcus pneumoniae, influenza), but few studies have examined the relationship with lifestyle related factors.

This study investigated the association between coffee and green tea intake, which are often drunk in Japan, and pneumonia in the elderly.

## Methods

### Study design

This hospital-based, matched case–control study was conducted at 24 hospitals in Tokyo, Chiba, Shizuoka, Aichi, Gifu, Kyoto, and Fukuoka Prefectures between October 1, 2009 and September 30, 2014. Details of this study have been described elsewhere^7–9^.

### Study subjects

As cases, the study included 65 years or older patients who were newly diagnosed with pneumonia by a physician. Pneumonia was diagnosed based on the increased white blood cell count (or elevated levels of C-reactive protein (CRP)), presence of an infiltrative shadow on chest X-rays, and clinical features (cough, sputum, and fever).

As a control, patients with the same sex and age (range of 5 years) who visited the same medical institution at the same time (within 2 months after examination of the case) for a disease other than pneumonia were selected. Two controls (respiratory department, other departments) for each case were selected whenever possible. Exclusion criteria were as follows: nursing home residents, patients with aspiration pneumonia (i.e., pneumonia caused by inhalation during eating or vomiting), patients with malignant tumors, patients currently undergoing treatment with oral steroids or immunosuppressant, and patients with a history of splenectomy.

### Information collection

The following information were collected from patient’s self-administered questionnaire and physician’s questionnaire: sex, age, height, body weight, vaccination status (pneumococcal, influenza), underlying diseases (respiratory disease, hypertension, diabetes mellitus, dyslipidemia, heart disease, cerebrovascular disease, kidney disease), activities of daily living (ADL), children ≤ 6 years old living in the same household, current smoking habit, current alcohol drinking habit, and coffee and green tea intake (how often and how much have you drunk for the past month?: Didn't you drink, how many cups did you drink a month, how many cups did you drink per week, how many cups did you drink a day?) (supplementary file).

### Statistical analysis

Explanatory variables were categorized into four groups for comparison as follows. Coffee (per day): did not drink, less than one cup, one cup, two or more cups, Green tea (per day): less than one cup, 1–2 cups, 3–4 cups, 5 or more cups.

Adjustment variables were classified as follows. Pneumococcal vaccination status was defined as "vaccinated" if a patient had undergone the vaccination within the previous 5 years, and "not vaccinated" if otherwise. Influenza vaccination (monovalent influenza A (H1N1) pdm09 vaccine, trivalent seasonal influenza vaccine) status was defined as "vaccinated" if the patient had undergone the vaccination within the previous 6 months, and "not vaccinated" if otherwise. During the 2009–2010 season, an influenza A (H1N1) pandemic occurred, but not a seasonal influenza epidemic^10^. The monovalent influenza A (H1N1) pdm09 vaccine was therefore used as the influenza vaccine. BMI was calculated as weight in kilograms (kg) divided by the square of height in meters (m^2^), and BMI was categorized as three groups according to WHO classification^11^; underweight (< 18.5 kg/m^2^); normal range (18.5–24.9 kg/m^2^); overweight (pre-obese (25.0–29.9 kg/m^2^) or obese (≥ 30.0 kg/m^2^)), and normal range was the reference category. All underlying diseases were categorized as yes, or no. ADL was categorized as "independent" or "not independent (bedridden, semi-bedridden, semi-independent)". Characteristics of cases and controls were compared using a Wilcoxon rank-sum test and chi-square, as appropriate.

We calculated the odds ratios (ORs) and 95% confidence intervals (CIs) for pneumonia using conditional logistic regression model to elucidate the association between coffee and green tea intake and pneumonia. The variables included in the multivariate model were factors that were p < 0.1 in the characteristic comparison of the cases and controls or were medically and biologically meaningful regardless of statistical significance. The following explanatory variables were included in the multivariate models: coffee and green tea intake, vaccination status (pneumococcal, influenza), BMI, underlying disease (respiratory disease, hypertension, diabetes mellitus, heart disease), ADL, children ≤ 6 years old living in the same household, current smoking habit, and current alcohol drinking habit. Trends for association were assessed by assigning ordinal scores to a single intake variable.

**Figure 1:**
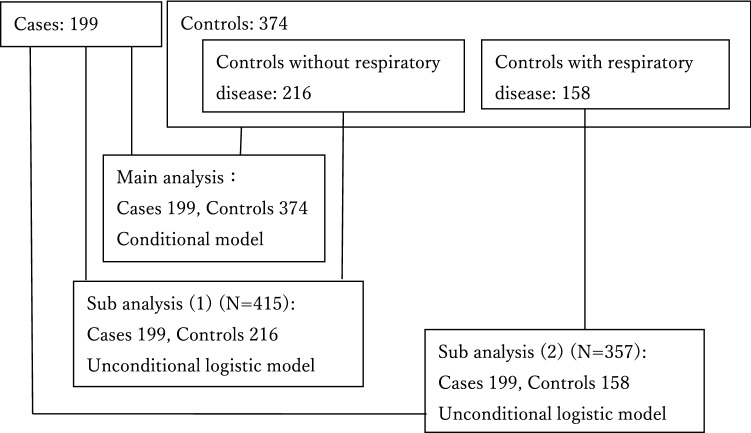
Subjects for analysis.

Next, following sub-analyses were performed. In our previous study examining control selection, different departments of control (respiratory department, other departments) showed different Vaccine inoculation rates, suggesting different background factors^8^. So when the characteristics were compared by control departments (respiratory department, other departments), there were patients who did not currently have respiratory disease but visited the respiratory department (52/192), and patients who currently have respiratory disease but visited other departments (18/182) (Table 1(a)). Therefore, as shown in Fig. 1, we investigated the association between coffee intake and pneumonia in following two subjects; Sub analysis (1): all cases (n = 199) and controls without respiratory disease (n = 216), Sub analysis (2): all cases (n = 199) and controls with respiratory diseases (n = 158). In two sub analyses, characteristics of cases (n = 199) and controls (n = 216 or n = 158) were compared using the same methods as the main analysis (Table 1(b)), the ORs of coffee intake for pneumonia were calculated using a logistic model.

**Table 1:** Comparison of characteristics.

Characteristic	Cases (N = 199)		Controls (N = 374)			(a) Controls by department				(b) Controls by underlying disease					
						Non- respiratory medicine (n = 182)		Respiratory medicine (n = 192)		Without respiratory disease (n = 216)			With respiratory disease (n = 158)		
	n	(%)	n	(%)	P*	n	(%)	n	(%)	n	(%)	P*^, †^	n	(%)	P*^, ‡^
**Age (years)**															
Median	75		75		0.570	74		75		74		0.205	76		0.622
Range	(65–92)		(65–98)			(65–93)		(65–98)		(65–93)			(65–98)		
Male sex	136	(68)	257	(69)	0.927	124	(68)	133	(69)	142	(66)	0.574	115	(73)	0.361
**Vaccinated**															
Pneumococcal vaccine	51	(26)	102	(27)	0.672	33	(18)	69	(36)	49	(23)	0.484	53	(34)	0.102
Influenza vaccine	86	(43)	181	(48)	0.237	82	(45)	99	(52)	98	(45)	0.659	83	(53)	0.080
**BMI (kg/m**^**2**^**)**															
< 18.5	49	(25)	47	(13)	< 0.001	15	(8)	32	(17)	22	(10)	< 0.001	25	(16)	0.017
18.5–24.9	121	(61)	246	(66)		121	(67)	125	(65)	147	(68)		99	(63)	
25–40	29	(15)	81	(22)		46	(25)	35	(18)	47	(22)		34	(22)	
**Underlying disease**															
Respiratory disease															
No	110	(55)	216	(58)	0.569	164	(90)	52	(27)	216	(100)		0	(0)	
Yes	89	(45)	158	(42)		18	(10)	140	(73)	0	(0)		158	(100)	
Hypertension	85	(43)	197	(53)	0.023	107	(59)	90	(47)	119	(55)	0.012	78	(49)	0.210
Diabetes mellitus	28	(14)	95	(25)	0.002	66	(36)	29	(15)	67	(31)	< 0.001	28	(18)	0.346
Dyslipidemia	31	(16)	70	(19)	0.348	43	(24)	27	(14)	50	(23)	0.052	20	(13)	0.434
Heart disease	29	(15)	77	(21)	0.078	51	(28)	26	(14)	51	(24)	0.020	26	(16)	0.625
Cerebrovascular disease	17	(9)	27	(7)	0.571	19	(10)	8	(4)	21	(10)	0.677	6	(4)	0.070
Kidney disease	4	(2)	15	(4)	0.203	6	(3)	9	(5)	8	(4)	0.304	7	(4)	0.226
**Activities of daily living (ADL)**															
Independent	176	(88)	347	(93)	0.080	172	(95)	175	(91)	204	(94)	0.028	143	(91)	0.530
Not independent	23	(12)	27	(7)		10	(5)	17	(9)	12	(6)		15	(9)	
**Live in a household with children ≤ 6 years old**	19	(10)	20	(5)	0.057	10	(5)	10	(5)	12	(6)	0.122	8	(5)	0.112
**Current smoker**	22	(11)	38	(10)	0.739	24	(13)	14	(7)	21	(10)	0.656	17	(11)	0.929
**Current alcohol drinker**	70	(35)	148	(40)	0.302	79	(43)	69	(36)	93	(43)	0.101	55	(35)	0.943

*Wilcoxon rank-sum test or Chi-square test or Fisher exact test.

^†^P between all cases (N = 199) and controls without respiratory disease (N = 216).

^‡^P between all cases (N = 199) and controls with respiratory disease (N = 158).

A difference was found in the comorbidities of diabetes mellitus in the characteristic comparison between the cases and the controls. Since coffee intake was often reported to have a protective association with diabetes mellitus^12,13^, the ORs of coffee intake for pneumonia in participants without diabetes mellitus were calculated using a logistic model (N = 450, sensitivity analysis).

In the sub-analyses and sensitivity analysis, matching could not be maintained, so an unconditional logistic model was used, and same variables as main analysis and matching variables (sex, age) were included in the multivariate models.

The level of statistical significance was taken as p < 0.05. Analysis was performed using SAS version 9.4 software (SAS Institute, Cary, NC).

### Ethics approval and consent to participate

The attending physician verbally explained the study using the written to the participants. Participants were informed that they could refuse to participate in this study and were instructed to complete a self-administered questionnaire if they could participant with this study, including providing medical information. All participants were considered to consent the study if they filled out the questionnaire. Informed consent was obtained from all participants. It is based on the research ethics guidelines for people in Japan at that time. This study protocol was approved by the Ethics Committees at the Osaka City University Graduate School of Medicine and was performed in accordance with the Declaration of Helsinki.

## Results

199 cases and 374 controls were enrolled. Table 1 shows characteristics of cases and controls. The subjects with BMI less than 18.5 kg/m^2^ were more frequent in cases than controls. The prevalence of hypertension and diabetes mellitus was significantly higher in the control than in the case. Other variables did not differ significantly between case and control.

Table 2 shows the association between coffee and green tea intake and pneumonia. ORs of coffee intake for pneumonia were 0.69 (95% CI 0.39–1.21) in those who drank less than one cup a day compared to those who did not drink coffee, 0.67 (0.38–1.18) in those who drank one cup, and 0.50 (0.28–0.88) in those who drank two or more cups, and the dose response relationship was significant (tend p = 0.024).

**Table 2:** Odds ratio of coffee and green tea intake for pneumonia.

	Cases		Controls		Crude OR	95% CI	P	Adjusted OR *	95% CI	P
	(N = 199)		(N = 374)							
	n	(%)	n	(%)						
**Coffee intake (daily)**										
None	52	(26)	66	(18)	1.00			1.00		
< 1 cup	49	(25)	89	(24)	0.68	0.40–1.14	0.139	0.69	0.39–1.21	0.192
1 cup	51	(26)	103	(28)	0.61	0.36–1.03	0.065	0.67	0.38–1.18	0.169
≥ 2 cups	47	(24)	116	(31)	0.49	0.29–0.83	0.008	0.50	0.28–0.88	0.017
					(Trend p = 0.010)			(Trend p = 0.024)		
**Green tea intake (daily)**										
< 1 cup	41	(21)	83	(22)	1.00			1.00		
1–2 cups	45	(23)	76	(20)	1.25	0.73–2.14	0.424	1.22	0.68–2.19	0.502
3–4 cups	65	(33)	130	(35)	0.998	0.59–1.69	0.995	1.18	0.67–2.05	0.571
≥ 5 cups	48	(24)	85	(23)	1.15	0.67–1.96	0.615	1.08	0.61–1.93	0.788
					(Trend p = 0.805)			(Trend p = 0.807)		

*Variables included in model: vaccination status (pneumococcal, influenza), BMI, underlying disease (respiratory disease, hypertension, diabetes mellitus, heart disease), ADL, children ≤ 6 years old living in same household, current smoking habit, current alcohol drinking habit, coffee intake, and green tea intake.

ORs of green tea intake for pneumonia were 1.22 (95%CI 0.68–2.19) in those who drank one or two cups a day compared to those who drank less than one cup, 1.18 (0.67–2.05) in those who drank three or four cups, and 1.08 (0.61–1.93) in those who drank five or more cups.

In Sub analysis (1), characteristics of BMI, prevalence of hypertension, diabetes mellitus and heart disease and ADL differed between the cases and the controls (Table 1(b)). As shown in Table 3, the OR of coffee intake for pneumonia was 0.49 (95% CI 0.26–0.93, p = 0.029) in those who drank two or more cups a day compared to those who did not drink coffee. In Sub analysis (2), only the BMI characteristics differed between the cases and the controls (Table 1(b)). The OR of those who drank two or more cups of coffee a day was 0.56 (0.30–1.07, 0.078) (Table 4).

**Table 3:** Odds ratio of coffee intake for pneumonia in Sub analysis (1).

	Cases		Controls without respiratory disease		Adjusted OR *	95% CI	P
	(N = 199)		(N = 216)				
	n	(%)	n	(%)			
**Coffee intake (daily)**							
None	52	(26)	34	(16)	1.00		
< 1 cup	49	(25)	57	(26)	0.60	0.32–1.11	0.105
1 cup	51	(26)	58	(27)	0.65	0.34–1.21	0.174
≥ 2 cups	47	(24)	67	(31)	0.49	0.26–0.93	0.029
					(Trend p = 0.054)		

*Variables included in model: sex, age, vaccination status (pneumococcal, influenza), BMI, underlying disease (hypertension, diabetes mellitus, heart disease), ADL, children ≤ 6 years old living in same household, current smoking habit, current alcohol drinking habit, coffee intake, and green tea intake.

**Table 4:** Odds ratio of coffee intake for pneumonia in Sub analysis (2).

	Cases		Controls with respiratory disease		Adjusted OR *	95% CI	P
	(N = 199)		(N = 158)				
	n	(%)	n	(%)			
**Coffee intake (daily)**							
None	52	(26)	32	(20)	1.00		
< 1 cup	49	(25)	32	(20)	0.98	0.51–1.87	0.943
1 cup	51	(26)	45	(28)	0.76	0.40–1.42	0.386
≥ 2 cups	47	(24)	49	(31)	0.56	0.30–1.07	0.078
					(Trend p = 0.055)		

*Variables included in model: same as Table 3.

In a sensitivity analysis of subjects without diabetes mellitus as an underlying disorder, the OR of those who drank two or more cups of coffee a day was 0.46 (95% CI 0.26–0.83, p = 0.010) compared to those who did not drink coffee (Table 5).

**Table 5:** Odds ratio of coffee intake for pneumonia excluding subjects with diabetes mellitus (sensitivity analysis) (N = 450).

Coffee intake (daily)	Cases		Controls		Sex and age adjusted OR	95% CI	P	Adjusted OR *	95% CI	P
	(N = 171)		(N = 279)							
	n	(%)	n	(%)						
None	48	(28)	55	(20)	1.00			1.00		
< 1 cup	42	(25)	66	(24)	0.71	0.41–1.24	0.231	0.74	0.42–1.33	0.315
1 cup	42	(25)	73	(26)	0.64	0.37–1.11	0.115	0.66	0.37–1.17	0.156
≥ 2 cups	39	(23)	85	(30)	0.50	0.28–0.87	0.014	0.46	0.26–0.83	0.010
					(Trend p = 0.014)			(Trend p = 0.010)		

*Variables included in model: sex, age, vaccination status (pneumococcal, influenza), BMI, underlying disease (respiratory disease, hypertension, heart disease), ADL, children ≤ 6 years old living in same household, current smoking habit, current alcohol drinking habit, coffee intake, and green tea intake.

## Discussion

We investigated the association between coffee and green tea intake and pneumonia among the elderly using hospital-based case–control study. Our study found a significant reduction in the OR for pneumonia in elderly individuals who drank ≥ 2cups/day of coffee compared to non-coffee drinkers. Our subjects without diabetes mellitus were also shown similar association.

Because in hospital-based case–control studies, it is desirable to select controls for different diseases to reduce bias, we recruited controls from the respiratory department and other departments. However, as a result of visiting a clinical department that has been examined in the past, there were patients who did not currently have respiratory disease but visited the respiratory department, and patients who currently have respiratory disease but visited other departments. So, when we performed the Sub analysis (1) in which the controls were limited to patients without respiratory disease and the Sub analysis (2) in which the controls were limited to patients with respiratory disease, the association between coffee intake and pneumonia were similar.

A large prospective cohort study in the United States showed an inverse association between coffee intake and total death, and there were inverse association between coffee intake and chronic respiratory diseases and pneumonia and influenza in deaths by cause^3^. Other cohort studies have also reported an inverse association between coffee intake and death from respiratory diseases (pneumonia, influenza, chronic obstructive pulmonary disease, and related symptoms)^4,14^. These findings suggest that coffee may have a preventive influence for chronic and acute respiratory diseases.

It has been reported that the constituents contained in coffee have various health benefits. There are several reports on the preventive association between coffee consumption and chronic respiratory disease and asthma^15^. Caffeine contained in coffee has arousal effect, inotropic effect, diuretic effect, and respiratory function improving effect, and theophylline of its metabolites, has bronchodilation, stimulation of respiratory center, and anti-inflammatory effect^16^. In addition, coffee components such as caffeine, chlorogenic acid, and trigonelline have been reported to have antibacterial activity^17–22^.

There are also some research reports on the association between coffee and intestinal flora. Mills CE and colleagues have tested in vitro that chlorogenic acid, a type of polyphenol abundant in coffee beans^23^, improves the balance of the gut flora^24^. In addition, arabinogalactan contained in coffee beans has an effect of growing specific bifidobacteria^25^, and bifidobacteria grown in the large intestine have a function of activating immune cells^26^. Because the intestinal flora changes with aging, for example the number of bifidobacteria that work well for the body reduce significantly after the age of sixty^27^, these coffee components may have a beneficial effect on the gut flora. The role of these components in coffee may have played a role in reducing the risk of pneumonia in the elderly seen in this study.

Green tea consumption was associated with a lower risk of death from pneumonia in Japanese women^28^. A Japanese cohort study reported that men who drank green tea had a reduced hazard ratio (HR) for respiratory disease-related mortality.^5^ We could not elucidate the association between green tea consumption and pneumonia. If caffeine was involved in the development of pneumonia, the amount of caffeine contained in 2 cups of coffee in this study is equivalent to 6 cups of green tea, so it is necessary to consider it in those who drink a lot of green tea (100 ml of beverage contains about 20 mg of caffeine for sencha and about 60 mg for regular and instant coffee^29^).

In the present study, the following limitations may have influenced the research results. First, information on intake of coffee and green tea was self-reported, and measurement of actual intake amount could not be made. Secondly, we didn't examine the detailed types of coffee and green tea that they ingested, so we could not investigate the association between the constituents of these drinks and pneumonia. The constituents of coffee differ depending on bean type, roasting method, instant, drip, or non-caffeine, and many kinds of tea are drunk in Japan, and the constituents are different depending on the type of green tea. Third, these drinking habits were information within the past month from the time of participation in this study, so the effects of long-term habits may not be considered. Because this study is hospital-based, controls may have changed their lifestyle within the past month due to their medical condition, and the possibility of reverse causality cannot be ruled out. Fourth, the possibility of confounding by associated factors that we did not examine is undeniable. For example, when examining the association between lifestyle and disease, socioeconomic status may affect lifestyle^30^, but we could not include it in our model as a potential confounder. Finally, because the study was conducted at 24 medical institutions in seven regions of Japan of different sizes, the severity of cases may vary between hospitals and clinics. Therefore, when a stratified analysis was performed at the hospital (with beds) and clinic (without beds), there was no difference in the association between coffee intake and pneumonia (Supplementary Table). However, since our study is a hospital-based case-contol study, in order to apply our result to the general elderly and the elderly with some diseases, further studies with more subjects and regions are needed.

## Conclusions

We examined the association between coffee and green tea intake and elderly pneumonia. A decrease in OR for pneumonia was suggested in those who drank more than two cups of coffee per day compared to those who did not. The intake of green tea was not related to pneumonia. Further studies are needed to clarify the association between coffee intake and pneumonia.

## Supplementary information

Supplementary Information.

Supplementary Table.

## Data Availability

The datasets used and/or analyzed during the current study are available from the corresponding author on reasonable request.

## Abbreviations

ADL: Activities of daily living
OR: Odds ratio
CI: Confidence interval
HR: Hazard ratio
