# Supplementary material for: Association between coffee and green tea intake and pneumonia among the Japanese elderly: a case-control study
Source: Sci Rep. 2021 Mar 10;11:5570. doi: 10.1038/s41598-021-84348-w (PMC7946905; doi:10.1038/s41598-021-84348-w)
Supplement: Supplementary file 1 — Supplementary Information. [file 41598_2021_84348_MOESM1_ESM.doc]

Association between coffee and green tea intake and pneumonia among the Japanese elderly: a case-control study

Kyoko Kondo^1^, *, Kanzo Suzuki^2, 3^, Masakazu Washio^4^, Satoko Ohfuji^5, 6^, Satoru Adachi^7^, Sakae Kan^8^, Seiichiro Imai^9, 10^, Kunihiko Yoshimura^11^, Naoyuki Miyashita^12^, Nobumitsu Fujisawa^13^, Akiko Maeda^5^, Wakaba Fukushima^5, 6^, Yoshio Hirota^14, 15^ and the Pneumonia in Elderly People Study Group

The Pneumonia in Elderly People Study Group is composed of:

Kanzo Suzuki^2, 3^, Masakazu Washio^4^, Kyoko Kondo^1^, Satoko Ohfuji^5, 6^, Akiko Maeda^5^, Wakaba Fukushima^5, 6^, Yoshio Hirota^14, 15^, Satoru Adachi^7^, Sakae Kan^8^, Seiichiro Imai^9, 10^, Kunihiko Yoshimura^11^, Naoyuki Miyashita^12^, Nobumitsu Fujisawa^13^, Noriko Kojimahara^16^, Chiharu Ota^17^, Ikuji Usami^17^, Munehiro Kato^17^, Toshinobu Yamamoto^17^, Kazuhide Yamamoto^18^, Yoichi Nakanishi^19^, Takanari Kitazono^19^, Takafumi Matsumoto^13^, Hideki Tashiro^13^, Masahiko Taketomi^20^, Tomoaki Iwanaga^21^, Hiroko Nogami^21^, Koichi Takano^22^, Ken Tonegawa^23^, Yoshimitsu Hayashi^24^, Ikuo Ikeda^25^, Shigeki Sugiyama^26^, Masahiro Aoshima^27^, Kei Nakashima^27^, Yoshitaka Nakamori^28^, Yasushi Seida^28^, Yoshiko Kichikawa^28^, Atsushi Nakamura^29^, Yasuhito Iwashima^30^, Yasuhiro Kojima^31^, Yasuo Yamada^32^, Hidekazu Kawamura^33^, Toshiaki Niwa^34^, Atsuro Kawai^35^ , Yuuji Ito^36^, Emi Aoyama^36^, Noriko Kusada^37^, Chizuko Sumida^37^

Author details

^1^ Osaka City University Hospital, Osaka, Japan

^2^ Department of Community-based Medical Education, Nagoya City University Graduate School of Medical Sciences, Nagoya, Japan

^3^ Nagoya City University, School of Nursing, Nagoya, Japan

^4^ Department of Community Health and Clinical Epidemiology, St. Mary's College, Kurume, Japan

^5^ Department of Public Health, Osaka City University Graduate School of Medicine, Osaka, Japan

^6^ Research Center for Infectious Disease Sciences, Osaka City University Graduate School of Medicine, Osaka, Japan

^7^ Department of Pulmonology, Kasadera Hospital, Nagoya, Japan

^8^ Kaisei Hospital, Nagoya, Japan

^9^ Department of Respiratory Medicine, Kyoto University Hospital, Kyoto, Japan

^10^ Preemptive Medicine and Lifestyle Related Disease Research Center, Kyoto University Hospital, Kyoto, Japan

^11^ Department of Pulmonology, Mitsui Memorial Hospital, Tokyo, Japan

^12^ Department of Internal Medicine, Kawasaki Medical School Hospital, Okayama, Japan

^13^ St. Mary’s Hospital, Kurume, Japan

^14^ Clinical Epidemiology Research Center, Medical Co. LTA (SOUSEIKAI), Fukuoka, Japan

^15^ College of Healthcare Management, Miyama, Japan

^16^ Tokyo Women’s Medical University, Tokyo, Japan

^17^ Asahi Rosai Hospital, Owariasahi, Japan

^18^ Kazu Clinic, Toyohashi, Japan

^19^ Graduate School of Medical Sciences, Kyushu University, Fukuoka, Japan

^20^ Doukai Clinic, Ookawa, Japan

^21^ Fukuoka National Hospital, Fukuoka, Japan

^22^ Nishifukuoka Hospital, Fukuoka, Japan

^23^ Nagoya City Koseiin Geriatric Hospital, Nagoya, Japan

^24^ Kasugai Municipal Hospital, Kasugai, Japan

^25^ Ikeda Clinic, Nagareyama, Japan

^26^ Sugiyama Clinic, Susono, Japan

^27^ Kameda Medical Center, Kamogawa, Japan

^28^ Mishuku Hospital, Tokyo, Japan

^29^ Nagoya City University Graduate School of Medical Sciences, Nagoya, Japan

^30^ Iwashima Clinic, Mizunami, Japan

^31^ Kojima Clinic, Nagoya, Japan

^32^ Yama Clinic, Aichi, Japan

^33^ Kawamura Clinic, Seki, Japan

^34^ Hamada Asai Clinic, Tajimi, Japan

^35^ Kawai Clinic, Osaka, Japan

^36^ Daiyukai Hospital, Ichinomiya, Japan

^37^ Inazawa Municipal Hospital, Inazawa, Japan

*Correspondence: kyou@med.osaka-cu.ac.jp

1 Osaka City University Hospital, Osaka, Japan

Contact address: Department of Public Health, Osaka City University Graduate School of Medicine, 1-4-3 Asahi-machi, Abeno-ku, Osaka 545-8585, Japan

Tel: +81-6-6645-3756; Fax: +81-6-6645-3757

|  |  |  | **‐** |  |  |  | **‐** |  |  |  | **‐** |  |
| --- | --- | --- | --- | --- | --- | --- | --- | --- | --- | --- | --- | --- |

（Please do not fill anything in this field）

**Survey on pneumonia prevention effect of influenza vaccine and pneumococcal vaccine**

**Questionnaire about lifestyle and living environment**

**Notes on entry**

・We will ask about the patient. Please fill in by yourself.

* The family can also fill it out.

・For each question, circle the applicable numbers or please write the answer in the underlined part.

・Please leave an empty space if you do not know how to fill it out.

Later, we will contact you by telephone from the following secretariat (Department of Public Health, Osaka City University Graduate School of Medicine).

・After filling in, please hand it to the doctor in charge. The contents of this questionnaire and medical information (test results, etc.) are totaled together.

- Those who cooperate with us will receive a 1,000 yen book card.

**Secretariat** Osaka City University Graduate School of Medicine Public Health

　　　　　　　Phone 06-6645-3756, 1-4-3 Asahi-cho, Abeno-ku, Osaka 545-8585, Japan

Entry date 　Heisei Year 　 　Month Day

Patient's name Sex 1. Male 2. Female

Date of birth Meiji/Taisho/Showa Year Month Day Age years

Person who filled in 1. Patient myself

2. Non-patients → Name

Relationship

Contact information in case of omission

Your name Your phone number

**Question 1.** I would like to ask you about the patient.

Height . cm

Weight . kg

**Question 2.** Where do you usually live?

1. Home 2. Hospital 3. Facility

◆ Number of family members (including patients) in the same household.

( ) person

◆ Do you live with children under 6 years old in the same household?

1. Yes 2. No

**Question 3.** Do you currently have a spouse?

1. Yes (including common-law marriage)

1. Living together 2. Separate

2. No 1. Divorced 2. Bereaved 3. Unmarried

**Question 4.** Which is the closest to your daily life?

| 1． | I spend all day on the bed. | I can't hit the roll over by myself. |
| --- | --- | --- |
| 2． |  | I can roll over and change my position. |
| 3． | I need caregiver help, either partially in the diet, in the toilet, or in my clothes, and spend most of my day on the bed. | I need assistance when transferring to wheelchairs, chairs, and portable toilets. |
| 4． |  | I can transfer to wheelchairs, chairs and portable toilets without assistance. |
| 5． | I can eat, use the toilet, and change clothes by myself, and spend a lot of time away from bed during the day. | I rarely go out if I have a caregiver. |
| 6． |  | I go out with assistance. |
| 7． | I can almost look after myself. | I go alone if I'm only in the neighborhood. |
| 8． |  | I go out by myself using transportation, etc. |

**Question 5.** Do you currently have the following illnesses?

Please circle as many as you like.

1. Hypertension 2. Dyslipidemia 3. Heart disease

4. Cerebral hemorrhage/cerebral infarction/stroke 5. Diabetes mellitus

6. Kidney disease 7. Others ( )

**Question 6.** Are you currently home oxygen therapy?

1. Yes 2. No

**Question 7.** Have you ever had the following respiratory diseases?

(If so, please indicate the age at which you were first diagnosed).

◆ Pulmonary emphysema  1．Yes (Around years old) 　2．No

◆ Chronic bronchitis　 　 1．Yes (Around years old) 　2．No

◆ Other chronic obstructive respiratory disease (COPD)

1．Yes (Around years old) 　2．No

◆ Pulmonary fibrosis 1．Yes (Around years old)　 2．No

◆ Bronchial asthma 　 　 1．Yes (Around years old) 　2．No

◆ pulmonary tuberculosis sequelae

1．Yes (Around years old) 　2．No

**Question 8.** Have you received the pneumococcal vaccine?

1．Yes　　　　　　　2．No　　　　　　3．Uncertain

◆ When did you inoculate?

　　　　　1．Within a year　　　2．(　　　)years ago　　　3．Uncertain

**Question 9.** Have you received the influenza vaccine in the last 6 months?

1．Yes　　 　2．No　　 　3．Uncertain

**Question 10.** How many times did you receive the influenza vaccine in the last three years?

Total ( ) times

**Question 11.** Does the patient currently have a habit of smoking?

1. I don't smoke.

2. I used to smoke, but I quit smoking. （　　　） years ago

3. I have a habit of smoking.

◆ Average number of pieces per day 　（　　　）pieces

◆ The age when I started smoking （　　　）years

**Question 12.** Do you currently have a habit of drinking alcohol?

1．I do not drink alcohol.

2．I used to drink alcohol, but I quit drinking alcohol.

3．I have a habit of drinking alcohol from time to time.

4．I have a habit of drinking alcohol every day.

◆ For the following drinks, tell how often and how much you took in the last month.

**Question 13.**  Coffee

1．I didn't drink

2．（　　　）cups per month

3．（　　　）cups per week

4．（　　　）cups per day

**Question 14.**　Black tea

1．I didn't drink

2．（　　　）cups per month

3．（　　　）cups per week

4．（　　　）cups per day

**Question 15.**  Green tea

1．I didn't drink

2．（　　　）cups per month

3．（　　　）cups per week

4．（　　　）cups per day

**That is all for questions. Thank you for your cooperation.**
