# Supplementary material for: Association between coffee and green tea intake and pneumonia among the Japanese elderly: a case-control study
Source: Sci Rep. 2021 Mar 10;11:5570. doi: 10.1038/s41598-021-84348-w (PMC7946905; doi:10.1038/s41598-021-84348-w)
Supplement: Supplementary file 2 — Supplementary Table. [file 41598_2021_84348_MOESM2_ESM.docx]

Association between coffee and green tea intake and pneumonia among the Japanese elderly: a case-control study

Kyoko Kondo^1^, *, Kanzo Suzuki^2, 3^, Masakazu Washio^4^, Satoko Ohfuji^5, 6^, Satoru Adachi^7^, Sakae Kan^8^, Seiichiro Imai^9, 10^, Kunihiko Yoshimura^11^, Naoyuki Miyashita^12^, Nobumitsu Fujisawa^13^, Akiko Maeda^5^, Wakaba Fukushima^5, 6^, Yoshio Hirota^14, 15^ and the Pneumonia in Elderly People Study Group

The Pneumonia in Elderly People Study Group is composed of:

Kanzo Suzuki^2, 3^, Masakazu Washio^4^, Kyoko Kondo^1^, Satoko Ohfuji^5, 6^, Akiko Maeda^5^, Wakaba Fukushima^5, 6^, Yoshio Hirota^14, 15^, Satoru Adachi^7^, Sakae Kan^8^, Seiichiro Imai^9, 10^, Kunihiko Yoshimura^11^, Naoyuki Miyashita^12^, Nobumitsu Fujisawa^13^, Noriko Kojimahara^16^, Chiharu Ota^17^, Ikuji Usami^17^, Munehiro Kato^17^, Toshinobu Yamamoto^17^, Kazuhide Yamamoto^18^, Yoichi Nakanishi^19^, Takanari Kitazono^19^, Takafumi Matsumoto^13^, Hideki Tashiro^13^, Masahiko Taketomi^20^, Tomoaki Iwanaga^21^, Hiroko Nogami^21^, Koichi Takano^22^, Ken Tonegawa^23^, Yoshimitsu Hayashi^24^, Ikuo Ikeda^25^, Shigeki Sugiyama^26^, Masahiro Aoshima^27^, Kei Nakashima^27^, Yoshitaka Nakamori^28^, Yasushi Seida^28^, Yoshiko Kichikawa^28^, Atsushi Nakamura^29^, Yasuhito Iwashima^30^, Yasuhiro Kojima^31^, Yasuo Yamada^32^, Hidekazu Kawamura^33^, Toshiaki Niwa^34^, Atsuro Kawai^35^ , Yuuji Ito^36^, Emi Aoyama^36^, Noriko Kusada^37^, Chizuko Sumida^37^

Author details

^1^ Osaka City University Hospital, Osaka, Japan

^2^ Department of Community-based Medical Education, Nagoya City University Graduate School of Medical Sciences, Nagoya, Japan

^3^ Nagoya City University, School of Nursing, Nagoya, Japan

^4^ Department of Community Health and Clinical Epidemiology, St. Mary's College, Kurume, Japan

^5^ Department of Public Health, Osaka City University Graduate School of Medicine, Osaka, Japan

^6^ Research Center for Infectious Disease Sciences, Osaka City University Graduate School of Medicine, Osaka, Japan

^7^ Department of Pulmonology, Kasadera Hospital, Nagoya, Japan

^8^ Kaisei Hospital, Nagoya, Japan

^9^ Department of Respiratory Medicine, Kyoto University Hospital, Kyoto, Japan

^10^ Preemptive Medicine and Lifestyle Related Disease Research Center, Kyoto University Hospital, Kyoto, Japan

^11^ Department of Pulmonology, Mitsui Memorial Hospital, Tokyo, Japan

^12^ Department of Internal Medicine, Kawasaki Medical School Hospital, Okayama, Japan

^13^ St. Mary’s Hospital, Kurume, Japan

^14^ Clinical Epidemiology Research Center, Medical Co. LTA (SOUSEIKAI), Fukuoka, Japan

^15^ College of Healthcare Management, Miyama, Japan

^16^ Tokyo Women’s Medical University, Tokyo, Japan

^17^ Asahi Rosai Hospital, Owariasahi, Japan

^18^ Kazu Clinic, Toyohashi, Japan

^19^ Graduate School of Medical Sciences, Kyushu University, Fukuoka, Japan

^20^ Doukai Clinic, Ookawa, Japan

^21^ Fukuoka National Hospital, Fukuoka, Japan

^22^ Nishifukuoka Hospital, Fukuoka, Japan

^23^ Nagoya City Koseiin Geriatric Hospital, Nagoya, Japan

^24^ Kasugai Municipal Hospital, Kasugai, Japan

^25^ Ikeda Clinic, Nagareyama, Japan

^26^ Sugiyama Clinic, Susono, Japan

^27^ Kameda Medical Center, Kamogawa, Japan

^28^ Mishuku Hospital, Tokyo, Japan

^29^ Nagoya City University Graduate School of Medical Sciences, Nagoya, Japan

^30^ Iwashima Clinic, Mizunami, Japan

^31^ Kojima Clinic, Nagoya, Japan

^32^ Yama Clinic, Aichi, Japan

^33^ Kawamura Clinic, Seki, Japan

^34^ Hamada Asai Clinic, Tajimi, Japan

^35^ Kawai Clinic, Osaka, Japan

^36^ Daiyukai Hospital, Ichinomiya, Japan

^37^ Inazawa Municipal Hospital, Inazawa, Japan

*Correspondence: kyou@med.osaka-cu.ac.jp

1 Osaka City University Hospital, Osaka, Japan

Contact address: Department of Public Health, Osaka City University Graduate School of Medicine, 1-4-3 Asahi-machi, Abeno-ku, Osaka 545-8585, Japan

Tel: +81-6-6645-3756; Fax: +81-6-6645-3757

**Supplementary Table.** Odds ratio of coffee intake for pneumonia stratified by institution

|  | | | Cases | | Controls | | Adjusted OR * | 95% CI | P |
| --- | --- | --- | --- | --- | --- | --- | --- | --- | --- |
| Hospital | | | (n = 156) | | (n = 288) | |  |  |  |
|  | Coffee intake (daily) | | |  |  |  |  |  |  |
|  |  | None | 41 | (26) | 50 | (17) | 1.00 |  |  |
|  |  | < 1 cup | 38 | (24) | 67 | (23) | 0.66 | 0.35-1.27 | 0.213 |
|  |  | 1 cup | 38 | (24) | 85 | (30) | 0.54 | 0.28-1.04 | 0.066 |
|  |  | ≥2 cups | 39 | (25) | 86 | (30) | 0.56 | 0.29-1.08 | 0.083 |
|  |  |  |  |  |  |  | (Trend p=0.077) | | |
| Clinic | | | (n = 43) | | (n = 86) | |  |  |  |
|  | Coffee intake (daily) | | |  |  |  |  |  |  |
|  |  | None | 11 | (26) | 16 | (19) | 1.00 |  |  |
|  |  | < 1 cup | 11 | (26) | 22 | (26) | 0.54 | 0.14-2.14 | 0.378 |
|  |  | 1 cup | 13 | (30) | 18 | (21) | 2.26 | 0.49-10.4 | 0.297 |
|  |  | ≥2 cups | 8 | (19) | 30 | (35) | 0.14 | 0.03-0.71 | 0.018 |
|  |  |  |  |  |  |  | (Trend p=0.070) | | |
| * Variables included in model: vaccination status (pneumococcal, influenza), BMI, underlying disease (respiratory disease, hypertension, diabetes mellitus, heart disease), ADL, children ≤6 years old living in same household, current smoking habit, current alcohol drinking habit, coffee intake, and green tea intake. | | | | | | | | | |
